# A Decaheme Cytochrome as a Molecular Electron Conduit in Dye-Sensitized Photoanodes

**DOI:** 10.1002/adfm.201404541

**Published:** 2015-03-11

**Authors:** Ee Taek Hwang, Khizar Sheikh, Katherine L Orchard, Daisuke Hojo, Valentin Radu, Chong-Yong Lee, Emma Ainsworth, Colin Lockwood, Manuela A Gross, Tadafumi Adschiri, Erwin Reisner, Julea N Butt, Lars J C Jeuken

**Affiliations:** School of Biomedical Sciences, University of LeedsLeeds, LS2 9JT, UK E-mail: L.J.C.Jeuken@leeds.ac.uk; The Astbury Centre for Structural Molecular Biology, University of LeedsLeeds, LS2 9JT, UK; Department of Chemistry, University of CambridgeLensfield Road, Cambridge, CB2 1EW, UK E-mail: Reisner@ch.cam.ac.uk; Advanced Institute for Materials Research, Tohoku University2–1–1 Katahira Aoba-ku Sendai, Miyagi, 980–8577, Japan E-mail: Hojo.Daisuke@wpi-aimr.tohoku.ac.jp; Centre for Molecular and Structural Biochemistry, School of Chemistry and School of Biological Sciences, University of East AngliaNorwich Research Park, Norwich, NR4 7TJ, UK E-mail: J.Butt@uea.ac.uk

**Keywords:** dye-sensitized nanoparticles, electron transfer, metalloprotein, photoelectrochemistry, protein-film electrochemistry

## Abstract

In nature, charge recombination in light-harvesting reaction centers is minimized by efficient charge separation. Here, it is aimed to mimic this by coupling dye-sensitized TiO_2_ nanocrystals to a decaheme protein, MtrC from *Shewanella oneidensis* MR-1, where the 10 hemes of MtrC form a ≈7-nm-long molecular wire between the TiO_2_ and the underlying electrode. The system is assembled by forming a densely packed MtrC film on an ultra-flat gold electrode, followed by the adsorption of approximately 7 nm TiO_2_ nanocrystals that are modified with a phosphonated bipyridine Ru(II) dye (RuP). The step-by-step construction of the MtrC/TiO_2_ system is monitored with (photo)electrochemistry, quartz-crystal microbalance with dissipation (QCM-D), and atomic force microscopy (AFM). Photocurrents are dependent on the redox state of the MtrC, confirming that electrons are transferred from the TiO_2_ nanocrystals to the surface via the MtrC conduit. In other words, in these TiO_2_/MtrC hybrid photodiodes, MtrC traps the conduction-band electrons from TiO_2_ before transferring them to the electrode, creating a photobioelectrochemical system in which a redox protein is used to mimic the efficient charge separation found in biological photosystems.

## 1. Introduction

As global energy consumption increases, the development of biological or bio-inspired systems for solar energy conversion has received intense interest.[1] Sunlight is the most abundant renewable energy source and is readily utilized by photosynthetic organisms through a complex cascade of photoinduced energy and electron-transfer steps, leading to efficient charge separation and high quantum yields.[2] The elegant way nature converts solar energy has inspired scientists to create synthetic photoelectrochemical cells in which biomimetic conversion systems generate electrical power or photofuels (ethanol, H_2_, etc.).[3,4] Many studies aim to integrate nature's light-harvesting systems such as chloroplasts,[5] thylakoids,[6,7] photosynthetic reaction centers of ­bacteria,[8–10] and ­photosystems (photosystems I and II)[11–13] with synthetic systems. In particular, by connecting these proteins to (semi-)conducting surfaces, bio-hybrid structures are formed capable of photochemistry.[14,15] A major drawback in many such photobioelectrochemical devices is the suboptimal orientation of the proteins on the surface, preventing fast electron transfer between the reaction center and the electrode, instead leading to nonproductive charge recombination.[16,17] Efforts have been made to improve the electron-transfer efficiency by embedding photosystem I and II in redox polymer-modified electrodes, which also enable control over the direction of electron transfer (cathodic vs anodic photocurrents).[11,18–20]

**Figure 1:**
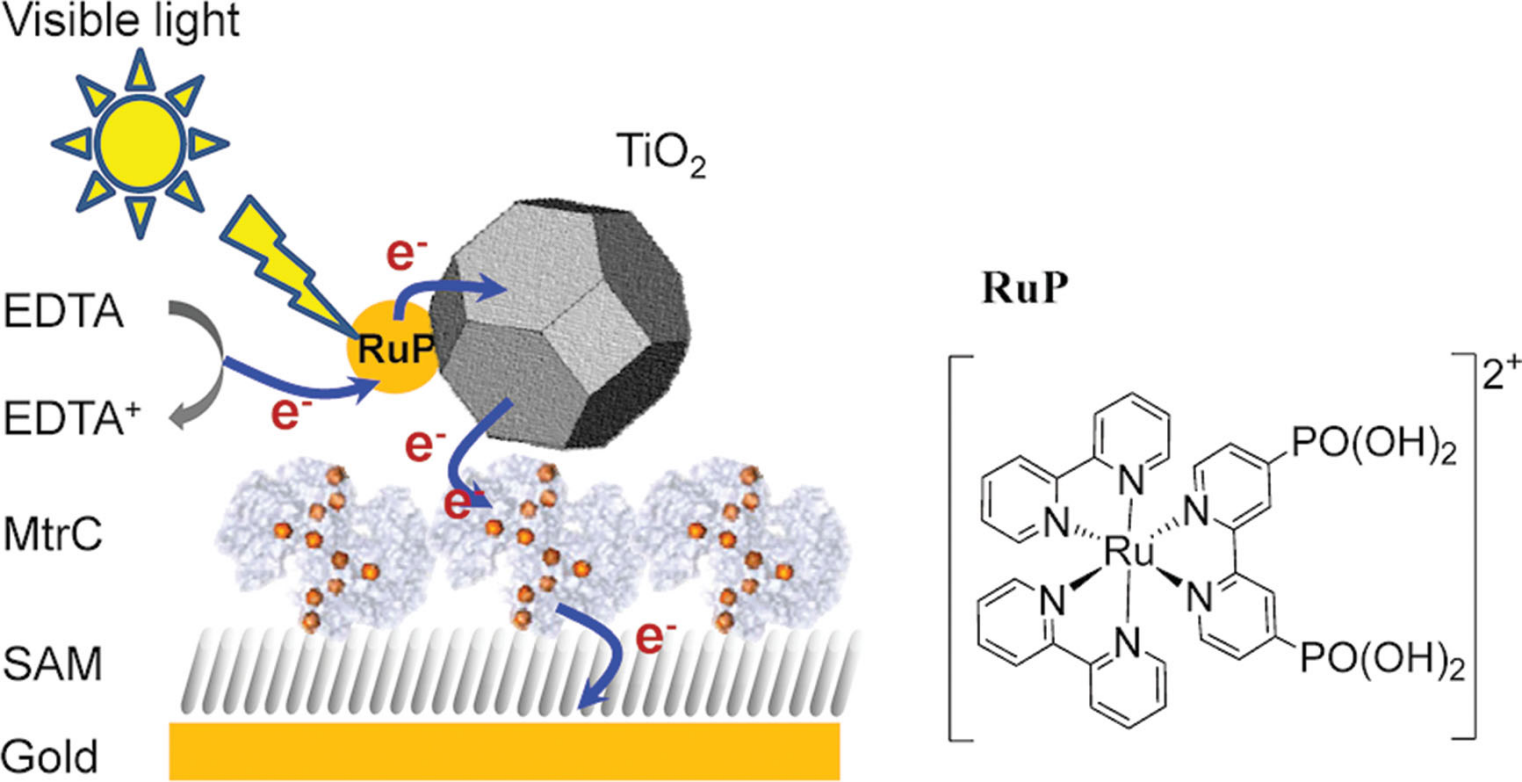
Left: Schematic representation of the layered MtrC/TiO_2_ system on a SAM-modified gold electrode and the interfacial electron-transfer steps required for the generation of a ­photocurrent. Right: The molecular structure of the cation Ru(bpy)_2_(4,4'-(PO_3_H_2_)_2_bpy)^2+^ (RuP).

Besides using biosystems for light harvesting, many studies have been performed with inorganic systems such as semiconducting nanoparticles or quantum dots.[21,22] Although inorganic materials do not suffer the generally short lifespan of their biological counterparts, they often suffer in their quantum yield as they are not as efficient in charge separation. In biology, charge separation proceeds by a series of fast electron-transfer steps along a chain or wire of redox centers. Here, we report on a hybrid system where we have coupled a dye-sensitized TiO_2_ nanocrystal to a decaheme protein from *Shewanella oneidensis* MR-1, known as MtrC (**Figure** **1**). In this biomimetic system, the dye-sensitized nanocrystal functions as the light-harvesting center and MtrC provides a long redox chain for charge separation. Electron transfer across the 10 hemes in MtrC will spatially separate the photoinduced charge from the nanocrystal, potentially reducing charge recombination. The successful construction of the biohybrid system is confirmed by a ­photoswitching behavior where the photocurrent depends on the redox state of the MtrC electrical bridge.

*Shewanella oneidensis* MR-1 is a dissimilatory metal-reducing bacterium, which can thrive in anaerobic environments by coupling the intracellular oxidation of electron donors and carbon sources to the respiratory reduction of a variety of extracellular electron acceptors, a feature that is extensively studied for exploitation in microbial electrochemistry.[23–27] Biochemical and structural studies on the outer-membrane proteins that are essential for extracellular respiration have led to a widely accepted porin-cytochrome model for the complexes that support electron transfer through the outer membrane (OM).[28,29] The porin-cytochrome complex consists of various multi-heme proteins capable of rapidly transferring electrons across tens of nanometers.[30] One of these proteins is the decaheme MtrC in which eight out of 10 hemes are organized in an almost linear chain, 7 nm in length.[31] MtrC lies at the extracellular face of the OM in *Shewanella oneidensis* MR-1[32] and is believed to operate at broad redox potentials that make it feasible to transfer electrons directly to extracellular insoluble mineral substrates with different redox potentials.[33]

To assemble the biomimetic photoelectrochemical cell schematically shown in Figure 1, we constructed hybrid layers of the molecular wire from MtrC and TiO_2_ nanocrystals photosensitized with a Ru(II)(bipyridine)_3_ dye in which one of the bipyridines is phosphonated in the 4,4'-position to enhance binding to TiO_2_ (RuP, see Figure 1 and Reisner et al.[34]). While negligible photocurrent response was observed using commercial P25 TiO_2_ nanoparticles (average crystallite size 21 nm, 8:2 ­mixture of anatase:rutile), greatly enhanced photocurrent was obtained using well-dispersed, in-house synthesized, anatase nanocrystals with diameters comparable to that of MtrC (6.8 ± 0.7 nm and approximately 3.5 nm × 6 nm × 7 nm, respectively). This novel MtrC/TiO_2_ system exhibits oxidative photocurrents that are dependent on the redox state of MtrC, thus confirming that electron transfer proceeds via MtrC. To the best of our knowledge, this report is the first demonstration of a photobioelectrochemical system that uses a redox protein to mimic efficient charge separation found in biological photosystems.

## 2. Results and Discussion

### 2.1. Synthesis and Characterization of Dye-Sensitized TiO_2_ Nanocrystals

**Figure 2:**
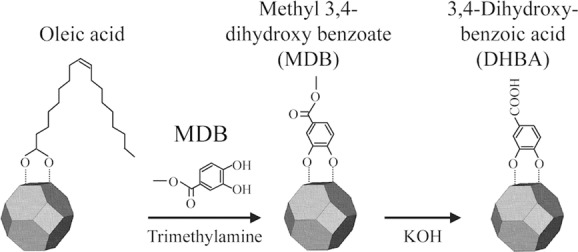
Scheme of the ligand-exchange reaction on the surface of oleic-acid-modified TiO_2_ nanocrystals to produce DHBA-TiO_2_ nanocrystals.

The TiO_2_ nanocrystals used for constructing the hybrid system were prepared by a high temperature solvothermal synthesis method to yield highly crystalline, oleic acid-capped (hydrophobic) particles with narrow size distribution. The particles were then transferred to the aqueous phase via a two-step ligand-exchange procedure using methyl-3,4-dihydroxy benzoate (MDB), which is subsequently converted to 3,4-dihydroxybenzoic acid (DHBA, **Figure** **2**). This two-step ligand exchange was used instead of direct modification with DHBA since both carboxylate and catechol groups can bind to TiO_2_; therefore, the ester group of MDB was used as a protecting group to ensure that the carboxylate group of DHBA was orientated away from the surface, resulting in highly water-dispersible nanoparticles. No change in crystallinity or particle size occurred during ligand exchange (transmission electron microscopy (TEM), X-ray diffraction (XRD)).

**Figure 3:**
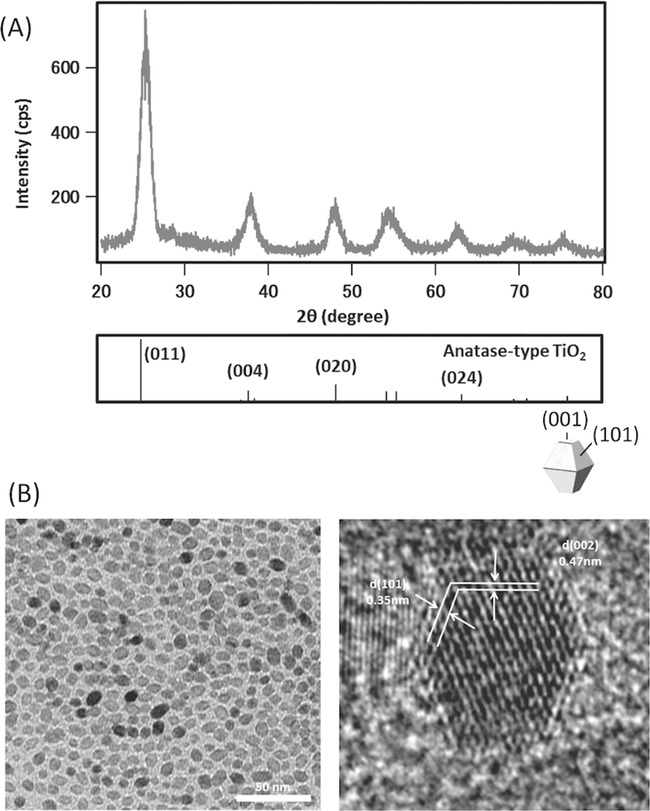
A) X-ray diffraction (XRD) pattern of 3,4-dihydroxybenzoic acid (DHBA)–TiO_2_ nanocrystals and bulk anatase TiO_2_ reference pattern (# JCPDS 84–1286) and B) transmission electron micrograph (TEM) images of oleic acid capped TiO_2_ nanoparticles.

The XRD pattern of the TiO_2_ nanoparticles showed a crystalline pure anatase phase (**Figure** **3**A) and, by TEM, the particles were found to be truncated octahedra with diameters of 6.8 ± 0.7 nm (Figure 3B). The organic content of the dried DHBA-TiO_2_ particles was measured to be 7.26% by thermogravimetric analysis, equating to ≈1.4 DHBA-molecules per square nanometer on the surfaces of the TiO_2_ nanocrystals (Figure S1, Supporting Information). The isoelectric point of the DHBA-TiO_2_, determined by zeta potential measurements (Figure S2, Supporting Information), was found to be 4.49, consistent with carboxylic acid functionality on the surface. Fourier-transform infrared spectroscopy (FT-IR) spectroscopy of the dried DHBA-TiO_2_ particles showed characteristic resonances for the DHBA modifier; however O–Ti–O peaks absorbances were too weak to be clearly observed (Figure S3-i, Supporting Information).

Dye-sensitization of the TiO_2_ nanocrystals was carried out by stirring the particles with an aqueous solution of RuP. The quantity of RuP bound to the TiO_2_ particles (herein referred to as RuP-TiO_2_) was estimated by UV–vis spectroscopy to be 90 ± 20 nmol mg^−1^. The RuP-TiO_2_ displayed characteristic phosphonate resonances in the FT-IR spectrum of a dried sample (Figure S3-ii, Supporting Information).

### 2.2. Construction and Characterization of the Photoanode

**Figure 4:**
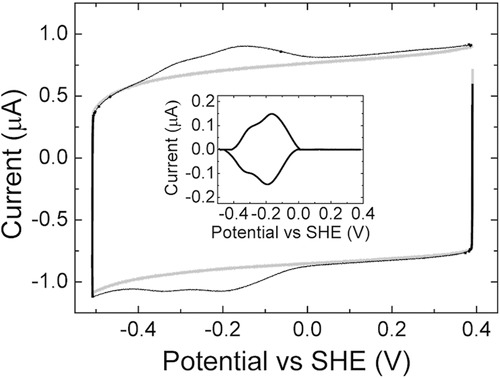
CV at 1 V s^−1^ and 20 °C before (gray) and after (black) adsorption of MtrC onto a 8-OH/8-NH_3_^+^ (ratio of 80/20) modified gold electrode in buffer (20 × 10^−3^ m MOPS, 30 × 10^−3^ m Na_2_SO_4_ at pH 7.4). The insert shows the baseline-subtracted protein signal.

To optimize construction of the photoanode composed of RuP-TiO_2_ and MtrC on a gold electrode, ultra-flat template-stripped gold surfaces were modified with a range of different self-assembled monolayers (SAMs), made up of various mixtures of alkanethiols with positive (amine), negative (carboxylic acid), and uncharged (hydroxyl) “headgroups.” A dilute MtrC protein solution (<1 × 10^−6^ m) was applied to the surface for 1 min at 20 °C after which non-bound MtrC was removed by rinsing with buffer. The purity of MtrC was confirmed with sodium dodecyl sulfate polyacrylamide gel electrophoresis (SDS-PAGE) and UV–vis electronic absorption spectroscopy (Figure S4, Supporting Information). Cyclic voltammetry (CV) was used to compare the electroactive coverage of MtrC on the various surfaces and the SAM composition was found to influence this property significantly. Redox signals were only detected with positively charged surfaces and the highest electroactive coverage was obtained with a SAM consisting of 8-mercaptooctanol (8-OH) and 8-amino-1-octanethiol (8-NH_3_^+^) in an 80/20 ratio (**Figure** **4**). At neutral pH, MtrC has an overall negative charge and the positively charged SAM is expected to interact favorably with the negatively charged propionate groups of the c-type hemes, facilitating the desired orientation in which MtrC can directly exchange electrons with the electrode.

Cyclic voltammograms of MtrC display reversible broad redox signals within a window of −0.4 to 0 V versus standard hydrogen electrode (SHE), similar to that previously published.[35,36] The complexity of the decaheme system makes it impossible to accurately determine interfacial electron-transfer rates, but even at scan rates of 1000 mV s^−1^, the oxidative and reductive peaks are fully reversible without any significant peak separation (Figure 4), indicating fast interfacial electron-transfer rates with *k*_0_ values above 100 s^−1^. Based on the peak area, the electroactive coverage (*⌈*_ea_) can be quantified according to *⌈*_ea_ = peak-area/*nFAυ*, where *n* is the number of electrons (10 for MtrC), *F* is the Faraday constant, *A* is the electrode area (0.25 cm^2^) and υ is the scan rate. The obtained electroactive coverage on the 8-OH/8-NH_3_^+^ surfaces is 0.56 ± 0.11 pmol cm^−2^.

**Figure 5:**
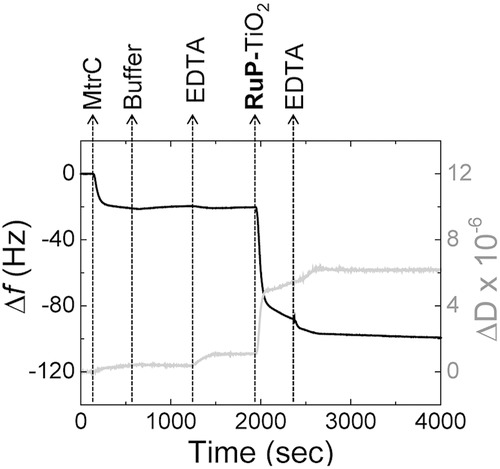
QCM-D results with frequency (black line, left axis) and dissipation (gray line, right axis) against time for a gold crystal at 21 °C. The gold surface is modified with 8-OH/8-NH_3_^+^ (ratio 80/20) SAM prior to the experiments. The plots shown are representative of triplicate experiments. As indicated, the gold crystal is consecutively incubated with: MtrC (0.87 × 10^−6^ m) in buffer (20 × 10^−3^ m MOPS, 30 × 10^−3^ m Na_2_SO_4_ at pH 7.4); buffer only; EDTA (25 × 10^−3^ m EDTA at pH 7.4); RuP-TiO_2_ (0.2 mg mL^−1^) in EDTA and, finally, EDTA.

The binding of MtrC onto the modified gold was further characterized using quartz-crystal microbalance with dissipation (QCM-D, **Figure** **5**), which provides information on both the mass and viscoelastic properties of the adsorbed protein layer through the frequency shift and the energy dissipation of the resonating crystal. MtrC adsorption saturates at −19 ± 1 Hz, which, according to the Sauerbrey equation, corresponds to ca. 0.34 ± 0.02 μg cm^−2^. Rinsing with buffer does not remove MtrC, indicating it is irreversibly adsorbed on the surface. Assuming the mass of MtrC is increased by about 25% due to water entrapped within the protein matrix,[37] the MtrC coverage can be estimated at 3.6 ± 0.2 pmol cm^−2^. The dimensions of MtrC are predicted to be 3.5 nm × 6 nm × 7 nm by comparison to the structure of the close homologue MtrF for which a crystal structure is available.[31] Depending on the orientation of MtrC on the surface, a closely packed monolayer would consist of 6.0 pmol cm^−2^ (upright orientation) or 3.0 pmol cm^−2^ (prone orientation).

**Figure 6:**
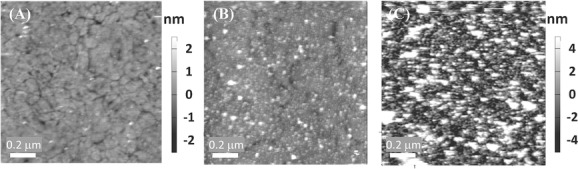
AFM height images of A) template stripped gold modified with a SAM, B) MtrC film, and C) MtrC/TiO_2_ system on the SAM-modified gold electrode. All images are 1 μm × 1 μm and displayed on a A) 5 or B,C) 10 nm z-scale. All surfaces were modified with SAM consisting of 8-OH/8-NH_3_^+^ (ratio of 80/20).

Comparing the electroactive coverage from CV (0.56 ± 0.11 pmol cm^−2^) with the total coverage from QCM-D, it is clear that only ≈15% of the adsorbed protein is electroactive. Presumably, the majority of the MtrC is adsorbed on the surface in a configuration that does not allow fast interfacial electron transfer. We note that the formation of a close-packed mono­layer of MtrC is required to prevent RuP**-**TiO_2_ from binding directly to the underlying gold electrode and thus for the formation of the biohybrid system. We thus complemented the CV and QCM-D studies with atomic force microscopy (AFM) to visualize the MtrC film. **Figure** **6**B shows typical AFM images of MtrC on the SAM surface, which indicate a dense mono­layer of MtrC. A few areas or spots are visible with heights above 4 nm, which seem to be consistent with the deposition of agglomerates of MtrC. Further analysis shows that these areas cover less than 5% of the surface. Further analysis also shows that the measured average height of the MtrC monolayer is 1.8 nm, about half the expected height (3.5 nm) for the prone orientation. This difference is consistent with a pressure-induced compression of the MtrC molecules, commonly observed with AFM.[38]

TiO_2_ has a high affinity for the carboxylates in Glu/Asp side chains and we previously used this propensity to couple P25 RuP-TiO_2_ nanoparticles to redox enzymes in solution.[34,39] However and unexpectedly, we did not observe immobilization of P25 TiO_2_ or RuP-(P25)TiO_2_ on either MtrC films or bare gold electrodes modified with SAMs (either pure or mixtures of 8-OH, 8-NH_3_^+^ and/or 8-mercapto-octanoic acid). To test whether the size of the nanoparticles has a major influence on its binding properties to the MtrC film, commercially obtained unmodified 5 nm anatase TiO_2_ particles were tested. However, also these smaller particles did not show any binding to the protein film, as tested with QCM-D.

In contrast, when we incubated the electrode surfaces with the here synthesized DHBA-modified TiO_2_ nanocrystals (0.2–0.5 mg mL^−1^), a densely packed TiO_2_ monolayer was formed on either MtrC films or bare SAM surfaces within 5 min, as monitored with QCM-D and AFM (Figure 6 and S5, Supporting Information). The reason for this stark contrast in behavior between the commercial nanoparticles and the DHBA-modified nanocrystals is suspected to be related to either differences in agglomeration tendency or surface chemistry of the particles. Modifying the P25 particles with DHBA and RuP did not affect the adsorption or photocurrent compared to unmodified P25.

QCM-D shows an additional frequency shift of ca. −80 ± 2 Hz and −120 ± 3 Hz for with and without MtrC, respectively. A low-to-moderate increase in dissipation is observed, indicating some dynamic flexibility of the interactions between RuP-TiO_2_ and the MtrC film or SAM surface. Based on the Sauerbrey equation, the frequency shifts ­correspond to 1.4 ± 0.1 μg cm^−2^ and 2.0 ± 0.1 μg cm^−2^ and, for TiO_2_ nanocrystals of 6.8 ± 0.7 nm diameter, we estimate the coverages to be 3.3 ± 0.1 pmol cm^−2^ and 4.8 ± 0.1 pmol cm^−2^ on the MtrC film and SAM surface, respectively. These values translate to ca. 66% and 96% of a theoretical monolayer coverage or, for the adsorption onto the MtrC film, a one-to-one complex between MtrC and TiO_2_. Following RuP**-**TiO_2_ adsorption, the electroactive coverage of MtrC decreases to 0.17 ± 0.03 pmol cm^−2^, reducing the percentage of MtrC that displays fast electron transfer with the electrode from 15% without RuP-TiO_2_ to 5% with RuP-TiO_2_. This suggests that the mobility and/or the orientation of MtrC is influenced by its interaction with the TiO_2_ nanocrystals. The AFM images of the films following RuP-TiO_2_ binding are consistent with close-to-monolayer coverages (Figure 6C and S5, Supporting Information) of TiO_2_ on top of MtrC, although some agglomerates are also visible. On the basis of the AFM images alone, it is difficult to distinguish between an MtrC monolayer, a TiO_2_ mono­layer, and/or a MtrC/TiO_2_ hybrid bilayer. To confirm the ­formation of an MtrC/TiO_2_ bilayer, the hybrid film was mechanically removed (i.e., scraped) from a small area by increasing the cantilever force during scanning. Subsequent imaging of a larger area indicated that the surface film has a thickness of ≈9 nm, consistent with an MtrC/TiO_2_ bilayer.

In the presence of ethylenediaminetetraacetic acid (EDTA) as a sacrificial electron donor, photocurrents are readily observed upon excitation of the RuP-TiO_2_ nanocrystals with visible light. Control experiments that lack one of the components (either RuP, TiO_2,_ or EDTA) gave no observable photocurrent. The absence of a photocurrent in the control experiment without TiO_2_ (but with 5 × 10^−3^ m RuP and 25 × 10^−3^ m EDTA) shows that RuP cannot directly donate electrons to MtrC and that the hemes in MtrC are not photoreduced by EDTA. Furthermore, in the presence of TiO_2_ (but absence of RuP), photocurrents were only observed under UV light, confirming that electron transfer proceeds from RuP via TiO_2_ to MtrC.

**Figure 7:**
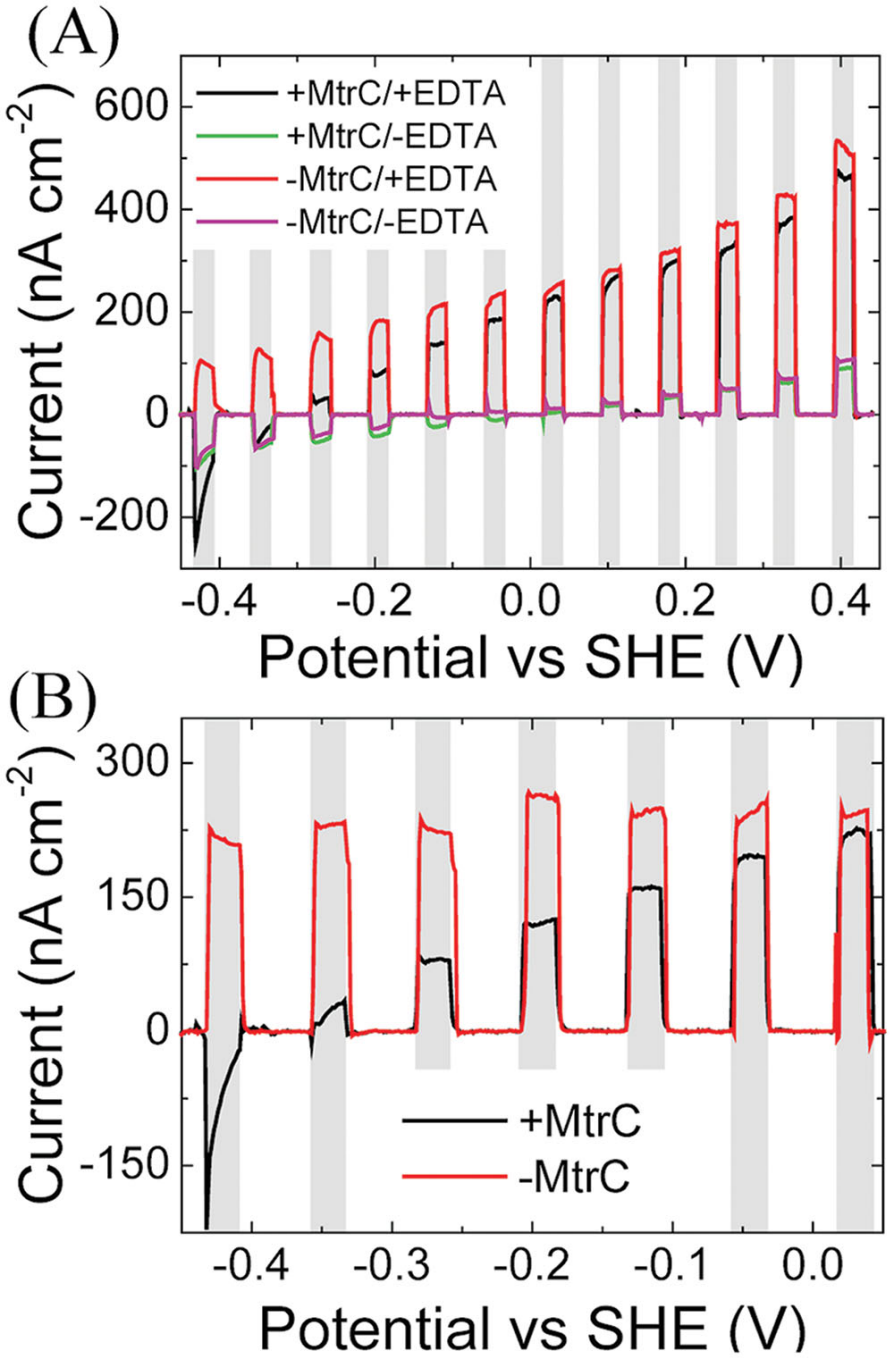
A) Effect of applied bias potential on the photocurrent of MtrC/RuP-TiO_2_ (+MtrC) and RuP-TiO_2_ only (−MtrC) measured with EDTA (+ EDTA) and without EDTA (−EDTA). The response was measured by linear sweep voltammetry (LSV) at 5 mV s^−1^. B) Normalized photocurrent (the difference between photocurrent generalized with and without the sacrificial electron donor EDTA). The gray bars represent the times during with the photoanodes are illuminated.

**Figure 8:**
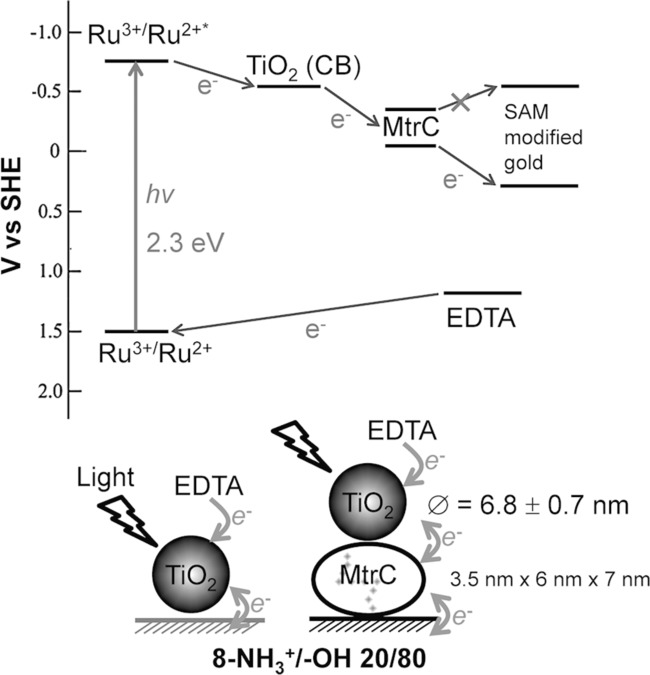
The predicted behavior of RuP-TiO_2_/MtrC photodiode based on the energy diagram.

Since MtrC is a redox protein that exists in oxidized and reduced states, it can also operate as an electrical diode or on/off switch for photocurrent. When MtrC is reduced at potentials below −0.3 V, no oxidative photocurrents are observed and the system behaves the same as without the sacrificial electron donor EDTA (**Figure** **7**A). This switch is clearly distinct from the behavior of RuP-TiO_2_ without MtrC (when TiO_2_ is directly adsorbed on the SAM-modified electrode). In Figure 7B, differences in photocurrent with and without EDTA are plotted, where the switch in oxidative photocurrent is more clearly visible upon reduction of MtrC. When MtrC is reduced (i.e., at potentials below 0 V), the electron transfer from RuP-TiO_2_ to MtrC is impaired, explaining the observed switch in photocurrent. The switch thus provides direct proof that for the MtrC/TiO_2_ hybrid system the majority of electron transfer proceeds via MtrC and that the densely packed monolayer of MtrC prevents direct interaction between the gold electrode and the RuP-TiO_2_ layer, confirming the formation of the layered structure schematically depicted in **Figure** **8**.

We have previously shown that fuel production by TiO_2_ particles coated with redox catalysts is limited by fast charge recombination.[40–42] This limitation is likely to apply to most inorganic photocatalytic systems where the catalyst is not ­separated from the light-harvesting particle. Here, we show that the decaheme MtrC can act as an electron-transfer conduit. Engineering a conduit-like MtrC between catalysts and TiO_2_ might reduce charge recombination by spatially separating the electron from the hole in the RuP-TiO_2_ nanoparticle thereby enhancing the efficiency of fuel production. Regretfully, in the hybrid MtrC/TiO_2_ system, oxidative photocurrents at +0.4 V are limited by light intensity up to 0.5 W cm^−2^ (Figure S6, Supporting Information), making it difficult to determine the rate-limiting electron-transfer step or to unambiguously determine whether charge recombination is reduced. Nonetheless, detailed analysis seems consistent with the idea that the MtrC conduit might improve photoefficiency.

Based on an electroactive coverage of 0.17 pmol cm^−2^ for MtrC, it can be calculated that, at 0.2 W cm^−2^ light intensity, 30 electrons per second are transferred through each electroactive MtrC. Based on the QCM-D data, it can be estimated that only 1 electron s^−1^ per RuP-TiO_2_ particle is generated for the photoanodes that do not contain a MtrC film. Thus, despite the fact that only 5% of the MtrC is electroactive and less RuP is adsorbed on the MtrC film than the anode without MtrC, the observed photocurrents are very similar between the RuP-TiO_2_ only and the RuP-TiO_2_/MtrC hybrid system.

In the MtrC/TiO_2_ hybrid system, four interfacial electron-transfer (ET) steps are required to obtain an oxidative photocurrent (see Figure 8): 1) from RuP to TiO_2_, 2) from TiO_2_ to MtrC, 3) from MtrC to the electrode, and 4) from the sacrificial electron donor, EDTA, to oxidized RuP. Electron transfer from RuP to TiO_2_ (step 1) occurs in the order of 180 ps, whereas recombination from TiO_2_ to oxidized RuP is much slower on the timescale of 1 ms.[39,41] We did not observe an increase in photocurrent upon increasing the EDTA concentration, suggesting that step 4 is also not rate limiting. The electron transfer from electroactive MtrC to the electrode (step 3) is less than 10 ms, i.e., much faster than the observed photo-generated 30 electrons s^−1^. By exclusion, it is thus possible that, besides light intensity, the electron transfer from TiO_2_ to MtrC (step 2) is rate limiting in the MtrC/TiO_2_ system.

A large fraction of the immobilized MtrC is not electroactive and RuP-TiO_2_ nanocrystals that are coupled to the nonelectroactive MtrC might thus not contribute to the photocurrent. To further study this, we added *N*,*N*'-dimethyl-4,4'-bipyridinium dichloride (methyl viologen, MV^2+^). MV^2+^ is an electron acceptor that has been shown to efficiently trap the conduction band electrons[43] and can efficiently transfer these trapped electrons directly to the underlying electrode. Indeed, addition of 1 × 10^−3^ m MV^2+^ leads to a fourfold enhancement in photocurrent (Figure S7, Supporting Information).

## 3. Conclusion

We have shown the assembly of a bio-hybrid system composed of a light-harvesting RuP-TiO_2_ nanocrystal and a molecular wire, the decaheme MtrC. Its formation is confirmed by the observed photoswitching behavior, which is due to the redox state of the MtrC electrical bridge on the electrode surface. At potentials below −0.4 V, in the fully reduced state of MtrC, no or very small cathodic photocurrents are observed. Upon increasing the applied potential, the (partly) oxidized MtrC acts as the initial electron acceptor for the photoexcited TiO_2_.

## 4. Experimental Section

*General Materials*: Unless otherwise stated, reagents and solvents were purchased from commercial suppliers and used without further purification: 3-(*N*-morpholino)propansulfonic acid (MOPS), 4-(2-hydroxyethyl)piperazine-1-ethanesulfonic acid (HEPES), sodium sulfate, EDTA disodium salt dehydrate, 8-mercaptooctanol, ethanol, and DHBA were purchased from Sigma–Aldrich (UK); titanium tetraisopropoxide, oleic acid, hexadecylamine, and methyl 3,4-dihydroxybenzoate were purchased from Wako Pure Chemical Industries Ltd; 8-amino-1-octanethiol hydrochloride was purchased from Dojindo Molecular Technologies; isopropanol and dichloromethane were purchased from Fisher Chemicals; methyl viologen hydrate was purchased from Acros Organics; EPOTEK 307 was purchased from Epoxy technology. [Ru(bpy)_2_(4,4'-(PO_3_H_2_)_2_bpy)](Br)_2_ (RuP) was prepared according to a literature procedure.[44] AEROXIDE P25 TiO_2_ particles were provided by Evonik Industries, and were a mixture of 8:2 anatase:rutile. Anatase 5 nm TiO_2_ particles were purchased from Nanostructure & Amorphous Materials, Inc.

*Synthesis of TiO_2_ Nanocrystals*: a) Oleic acid-capped TiO_2_: The precursor, 1 mmol of titanium tetraisopropoxide and the modifiers, 3 mmol of oleic acid and 0.5 mmol of hexadecylamine were heated at 120 °C for 5 min as a pretreatment and the mixture was placed in a pressure-resistant Hastelloy vessel (inner volume = 5 mL) with a Hastelloy sphere. This resulted in a well-mixed solution of the precursor and modifiers. Next, the solvothermal reaction was made to occur in the reactor at 400 °C for 10 min. The organic ligand-modified nanocrystals were extracted from the product mixture using cyclohexane (3 mL). The products were precipitated from the resulting cyclohexane phase using ethanol (3 mL) as an antisolvent agent and were separated using centrifugation. The centrifuged products were then washed twice with a mixture of cyclohexane (3 mL) and ethanol (3 mL). The washed products were dissolved in cyclohexane (5 mL). b) Ligand exchange: A solution mixture containing 25 mg of methyl 3,4-dihydroxybenzoate (MDB) and ethanol (2 mL) was delivered dropwise into cyclohexane dispersing the oleic acid-modified TiO_2_ nanocrystals (2 mL, 0.7 wt%) and the mixtures were stirred at the room temperature for 2 h after introducing trimethylamine (200 mg). The catechol groups of MDB react with the surfaces of TiO_2_ nanocrystals exchanging the oleic acid. The MDB-modified TiO_2_ nanocrystals were centrifuged to separate them from residual MDB and oleic acid. The centrifuged products were then washed with a mixture of cyclohexane (4.5 mL) and ethanol (4.5 mL) and centrifuged once more. The washed pellet was dispersed in water (9 mL) adding 50 μL KOH aqua (5 m) and the mixtures were stirred for 30 min, followed by ultrasonic treatment for 30 min so that ester bond in MDB hydrolyzed. The products were centrifuged after adding acetone (5 mL) and the centrifuged products were then washed with a mixture of acetone (5 mL) and water (4 mL). After centrifugation, the washed products were dispersed in water (4 mL) to produce a DHBA–TiO_2_ nanocrystals dispersion. c) Attachment of RuP: RuP (1 × 10^−3^ m, aq.) was added to the solution of DHBA–TiO_2_ in a ratio of 1 μmol RuP per 5 mg nanocrystals. The solution was allowed to mix for at least 1 h before use, and was stored protected from light. To analyze the extent of RuP attachment, the nanoparticles were precipitated by acidification (0.1 m HCl) and centrifuged (10 000 rpm, 5 min). Comparison of the absorbance at 457 nm of the supernatant to a reference solution gave an estimate of 0.09 ± 0.02 μmol RuP per mg particles. Acidification of RuP alone did not result in any precipitation.

*DHBA Functionalization and Dye-Sensitization of P25 TiO_2_*: P25 TiO_2_ (20 mg) was suspended in acetone (200 mL) with sonication for 10 min. DHBA (20 mg) was added and the mixture was stirred for 5 min. The particles were isolated by centrifugation (4000 rpm, 5 min), washed with acetone, and re-isolated by centrifugation. The particles were allowed to dry in air, protected from light.RuP (1 × 10^−3^ m, aq.) was added to a suspension of DHBA-(P25)TiO_2_ in water, in a ratio of 0.2 μmol RuP per 5 mg nanoparticles. The solution was allowed to mix for 1 h, protected from light. The particles were isolated by centrifugation (4000 rpm, 5 min) and allowed to dry in air, protected from light. Comparison of the optical absorbance at 457 nm of the supernatant from centrifugation, relative to a reference solution, gave an estimate of 0.03 μmol RuP per mg particles.

*Characterization of RuP-TiO_2_ Nanocrystals*: TEM (JEM-2100F, JEOL) was used for high-resolution image at 200 keV. XRD patterns were obtained with a Rigaku D/Max-2500 diffractometer using Cu Kα radiation (*λ* = 1.5418 Å) at a scanning rate of 4.00 degrees min^−1^. Zeta potential measurements were carried out using a Malvern Instruments nanocomposite size analyzer (NanoZS, Worcestershire, UK). FT-IR spectra were obtained using a Thermo Scientific Nicolet iS50 FTIR spectrometer in ATR mode.

*Characterization of MtrC*: A soluble form of MtrC was purified as described in Edwards et al. (under review) from a construct in which the 25 N-terminal amino acids of native MtrC are replaced with the signal sequence of MtrB from *S. oneidensis* MR-1 (MKFKLNLITLALLANTGLAVAADG). The resulting MtrC sample (87 × 10^−6^ m MtrC in 20 × 10^−3^ m HEPES, pH 7.5) was subject to SDS-PAGE followed by protein visualization using Coomassie Brilliant Blue staining (Figure S3, Supporting Information). A single protein band was revealed with an apparent molecular mass of 75 kDa as expected for MtrC and confirming that the protein was pure (>95%). Electronic absorbance spectroscopy of the purified MtrC confirmed the presence of c-type hemes (Figure S4, Supporting Information). The spectrum of the air-oxidized MtrC is characterized by a prominent band in the Soret (γ) region with a maximum at 410 nm and accompanied by smaller features at 531 and 560 nm. MtrC reduced by sodium dithionite displays a spectrum typical of low-spin ferric c-heme with a peak maximum at 419 nm, together with clearly defined α- and β-peaks at 552 and 523 nm, respectively.

*Electrode Preparation and MtrC Film Voltammetry*: The template-stripped gold (TSG) as working electrodes was prepared by a method described previously.[45] Using an Edwards Auto 306, 150 nm gold (99.99%; Goodfellow) was evaporated on silicon wafers (IDB Technology Ltd, UK). After evaporation, 1.2 cm^2^ glass slides were glued to the gold layer with Epo-Tek 377 for 2 h at 120 °C. SAMs were made by incubating the detached glass slide, exposing the TSG surface, with 0.8 × 10^−3^ m 8-OH/0.2 × 10^−3^ m 8-NH_3_^+^ in ultrapure water (Milli-Q water, 18.2 MΩ cm) for more than 2 days at 4 °C. After incubation, the excess thiol was gently washed away with Milli-Q water and then dried with nitrogen. For the protein film electrochemistry experiment, a home-build electrochemical cell was used with a standard three-electrode setup. As the working electrode, the SAM-modified TSG was embedded in a polytetrafluoroethylene (PTFE) holder with a rubber O-ring seal, placed in a glass electrochemical cell container with a platinum wire counter electrode and a saturated silver/silver chloride reference electrode (Ag/AgCl; Radiometer analytical, France) and 2 mL of buffer (20 × 10^−3^ m MOPS, 30 × 10^−3^ m Na_2_SO_4_ at pH 7.4) was added. All potentials are quotes versus SHE using 0.199 mV vs SHE for the Ag/AgCl reference electrode. The quality of the SAM was assessed with electrochemical impedance spectroscopy spectra before the immobilization of MtrC. To form the MtrC protein film, the electrolyte was removed and the electrode was submersed in 50 μL of protein solution (0.87 × 10^−6^ m) for 1 min at 20 °C. After rinsing the electrochemical cell more than three times with 2 mL buffer making sure the electrode remains under fluid throughout, cyclic voltammograms (CVs) were measured in fresh buffer (2 mL). To immobilize RuP-TiO_2_ nanocrystals, the electrolyte solution was almost completely removed and 50 μL RuP-TiO_2_ nanocrystal or P25 suspension (typically 02–0.5 mg mL^−1^) was added. The nanocrystal solution was freshly diluted in a 25 × 10^−3^ m EDTA solution (pH 7.4), as prolonged storage in EDTA or buffer solutions was observed to result in agglomeration of the nanocrystals. After incubation for 5 min, the electrochemical cell was rinsed several times with 20 × 10^−3^ m EDTA to remove any non-bound RuP-TiO_2_ nanocrystals. CVs were obtained using an Autolab electrochemical analyzer (Ecochemie, Utrecht, Netherlands) equipped with a PGSTAT 128N potentiostat, SCANGEN and ADC750 modules, and FRA2 frequency analyzer (Ecochemie). CV experiments were routinely carried out by holding the potential at 0.19 V for 5 s before cycling at a scan rate of 1 V s^−1^ in the potential window from 0.4 V to −0.5 mV (vs SHE). Voltammograms were analyzed using the freely available software Q-Soas.[46] The electrochemical cell was used in a steel mesh Faraday cage to minimize electrical noise, and all experiments were conducted under purging with argon.

*Quartz Crystal Microbalance with Dissipation*: QCM-D measurements were performed using a Q-Sense E4 (Q-Sense AB). Prior to each measurement the gold-coated QCM-D crystals (Q-Sense AB) were cleaned with 2 wt% SDS detergent for 10 min in a bath sonication, rinsing with Milli-Q water and dried under a nitrogen flow. QCM-D crystals were subsequently treated for 20 min with UV/ozone (UVOCS Inc T10 × 10/OES/E, UK) followed by a 30 min reduction in freshly distilled propanol at 40–60 °C using a Soxlet setup. Freshly cleaned QCM-D crystals were modified with a SAM as above and then rinsed with Milli-Q water. QCM-D experiments were conducted at 21 °C, with the flow rate held at 71 μL min^−1^. Incubations with MtrC (0.87 × 10^−6^ m) and RuP-TiO_2_ (0.2 mg mL^−1^) are performed as indicated in the result section. All protein-binding experiments were performed in buffer (20 × 10^−3^ m MOPS, 30 × 10^−3^m Na_2_SO_4_ at pH 7.4). On graphs, changes in the dissipation (Δ*D*) and frequency (Δ*f*) of the third overtone are presented, while fifth, seventh, ninth, eleventh, and thirteenth overtones were also recorded.

*Atomic Force Microscopy*: For AFM images, TSG surfaces were prepared and modified as described for the electrochemistry experiments. All AFM images were taken using an Asylum Research MFP-3D-SA atomic force microscope, operated in intermittent contact mode, using SNL cantilevers (Bruker) having nominal spring constants of 0.24 N m^−1^ and nominal tip radii of 2 nm. Free oscillation amplitudes were ca. 15 nm and the amplitude set point adjusted to maintain imaging forces of 150–250 pN. Scan rates were 1–2 Hz.

*Development and Photoelectrochemical Measurement of TiO_2_/MtrC Conduit Wire*: Photoelectrochemical measurement were performed using a cold light source featuring a 150 W, 4.5 cm (15 V) halogen lamp (OSRAM) with a fiber optic arm (Krüss KL5125, Germany). The light source was placed 5–10 cm above the gold electrode with the light passing through ≈2 cm of buffer before reaching the electrode surface. The light intensity on the sample (of 0.25 cm^2^) was approximately 50 mW. The UV filter ensured that no UV excitation of the TiO_2_ was possible. The photoinduced currents were measured between the modified working electrodes and a Pt counter electrode using Autolab electrochemical analyzer, as above. Linear scan voltammetry (LSV) was used to determine the photoelectrochemical properties of RuP–TiO_2_ nanoparticles and TiO_2_/MtrC conduit system under controlled illumination. The measurement was performed at a scan rate of 5 mV s^−1^ between −0.45 and +0.45 V versus SHE. For chronoamperometry measurements, the potential *E* = +400 mV versus SHE was set and an illumination was typically set to 10 s (40 s off period). Measured photocurrents are after baseline correction (during the dark phases) with Q-Soas.

## Supporting Information

As a service to our authors and readers, this journal provides supporting information supplied by the authors. Such materials are peer reviewed and may be re-organized for online delivery, but are not copy-edited or typeset. Technical support issues arising from supporting information (other than missing files) should be addressed to the authors.
