# Supplementary material for: A Decaheme Cytochrome as a Molecular Electron Conduit in Dye-Sensitized Photoanodes
Source: Adv Funct Mater. 2015 Mar 11;25(15):2308–15. doi: 10.1002/adfm.201404541 (PMC4493899; doi:10.1002/adfm.201404541)
Supplement: Supplementary file 1 — Supplementary [file adfm0025-2308-sd1.pdf]

Copyright WILEY-VCH Verlag GmbH & Co. KGaA, 69469 Weinheim, Germany, 2015.

ADVANCED FUNCTIONAL MATERIALS

Supporting Information

for *Adv. Funct. Mater.*, DOI: 10.1002/adfm.201404541

A Decaheme Cytochrome as a Molecular Electron Conduit in Dye-Sensitized Photoanodes

Ee Taek Hwang, Khizar Sheikh, Katherine L. Orchard, Daisuke Hojo, Valentin Radu, Chong-Yong Lee, Emma Ainsworth, Colin Lockwood, Manuela A. Gross, Tadafumi Adschiri, Erwin Reisner,* Julea N. Butt,* and Lars J. C. Jeuken**

ASSOCIATED CONTENT

A Decaheme Cytochrome as a Molecular Electron Conduit in Dye-Sensitized Photoanodes

Ee Taek Hwang,[†,‡] *Khizar Sheikh,*[†,‡] *Katherine L. Orchard,*[§,∥] *Daisuke Hojo,*[*,∥] *Valentin Radu,*[†,‡] *Chong-Yong Lee,*[§] *Emma Ainsworth,*[±] *Colin Lockwood,*[±] *Manuela A. Gross,*[§] *Tadafumi Adschiri,*[∥] *Erwin Reisner,*[*,§] *Julea N. Butt,*[*,±] *and Lars J. C. Jeuken**[†,‡]

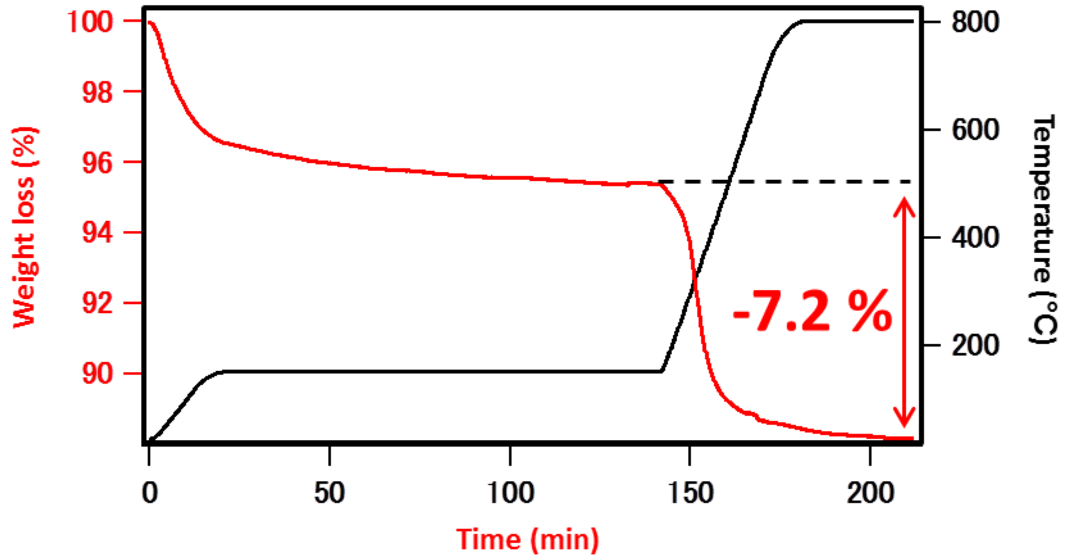

Figure S1. Thermogravimetric analysis (TGA) of DHBA-TiO_2 nanoparticles.

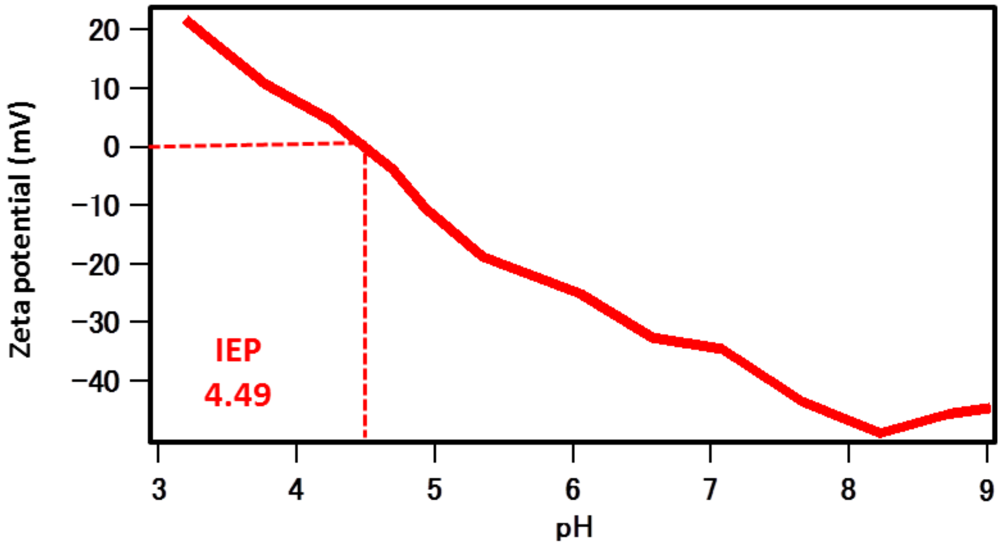

Figure S2. Zeta potential of DHBA-TiO_2 as a function of pH. The isoelectric point (IEP) is highlighted.

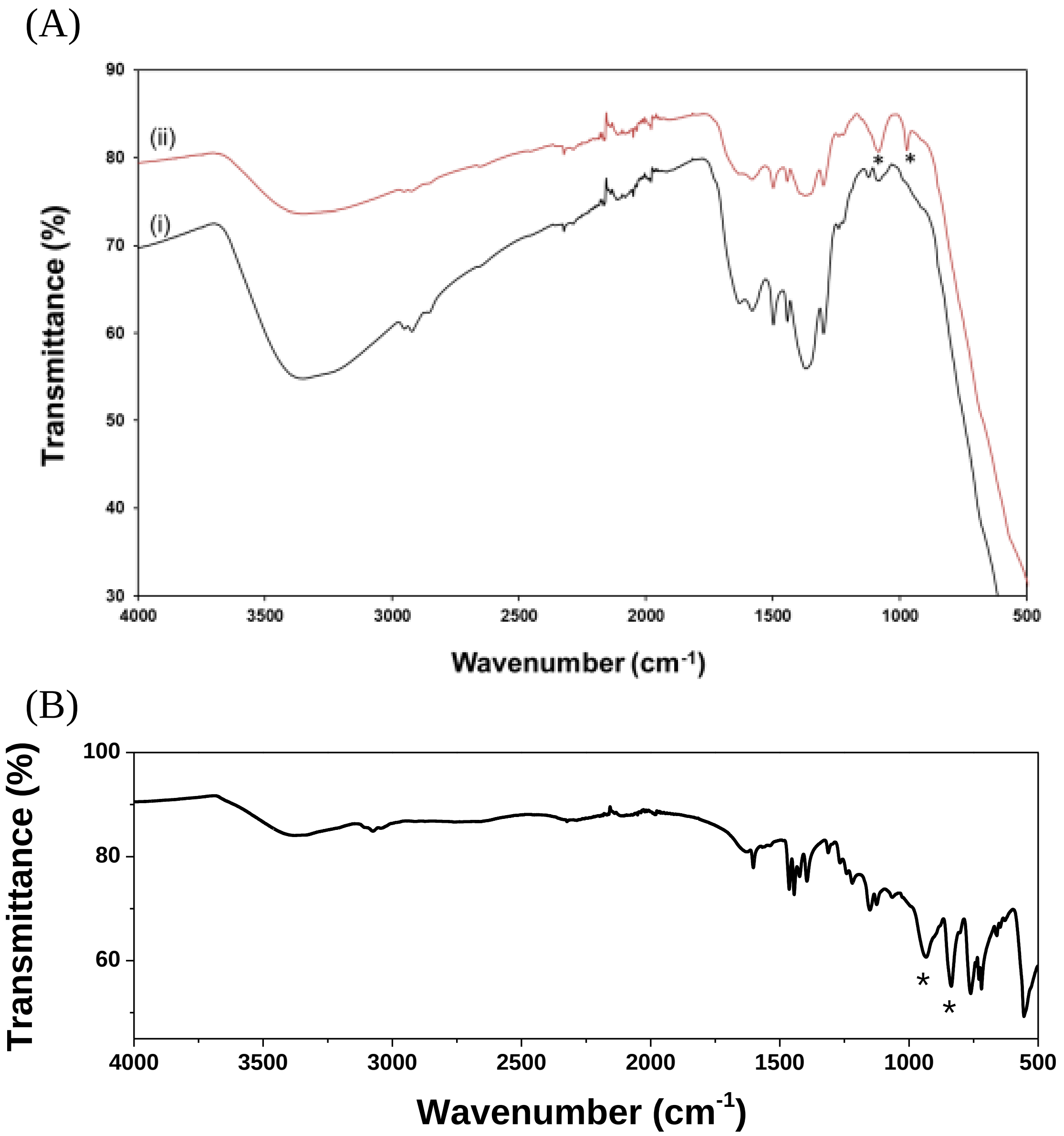

Figure S3. Fourier-transform infrared (FT-IR) spectra of (A) (i) DHBA-TiO_2 nanocrystal and (ii) **RuP**-DHBA-TiO_2 and (B) **RuP**; Peaks marked with “*” correspond to the phosphonate groups of **RuP**.

(A)

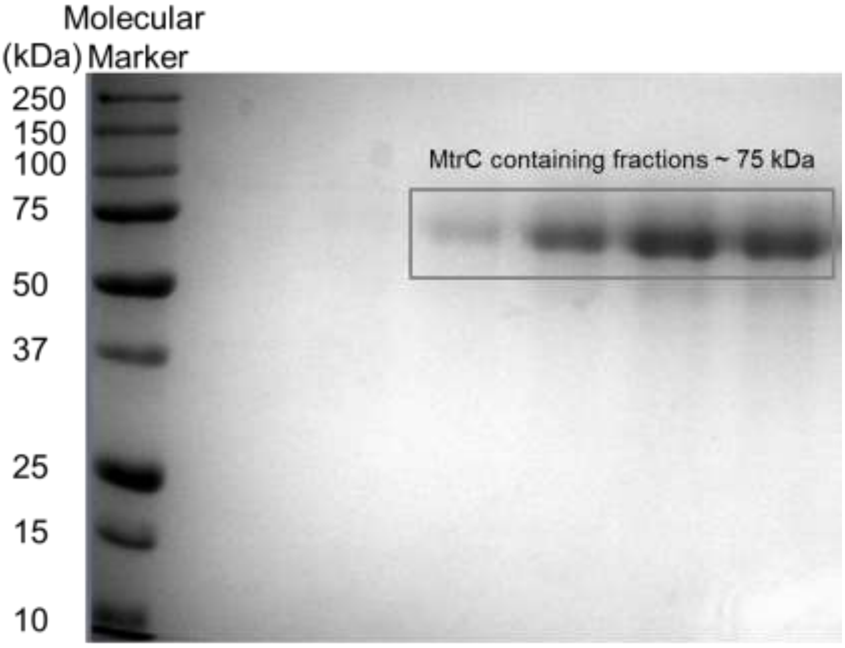

(B)

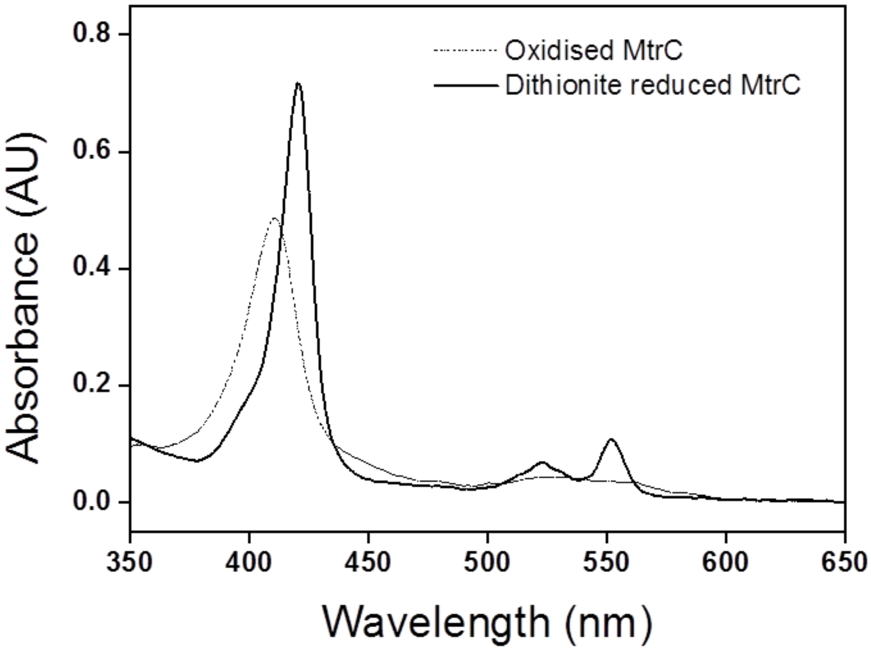

Figure S4. Characterization of MtrC protein (A) SDS/PAGE analysis. The proteins were resolved by 12% (wt/vol) SDS/PAGE and stained with Coomassie blue (B) Electronic absorbance spectra of MtrC, Oxidized (dash line) and sodium dithionite reduced (solid line).

(A)

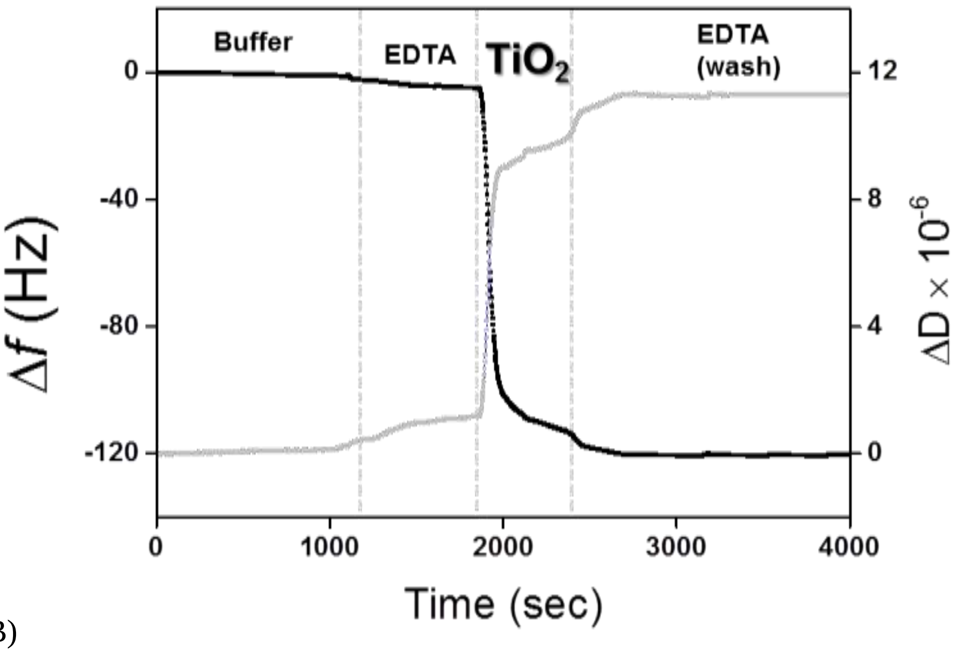

(B)

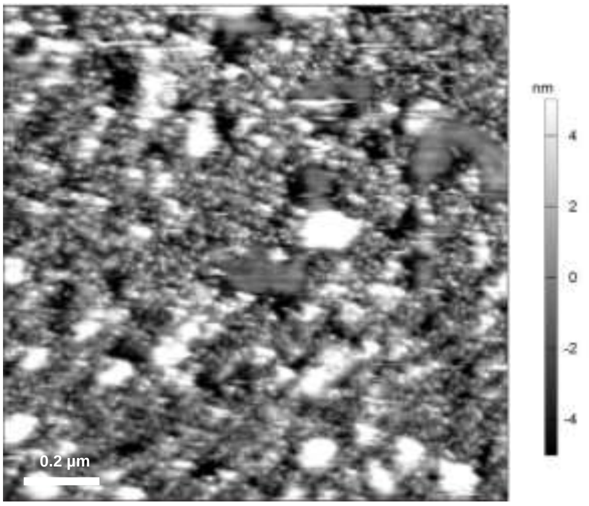

Figure S5. Characterizations of **RuP**-TiO_2 nanocrystals on SAM-modified gold surfaces (A) QCM-D results in buffer (20 mM MOPS, 30 mM Na_2SO_4 at pH 7.4) with frequency (black line, left axis) and dissipation (grey line, right axis) against time. To measure absorbed nanocrystals on electrode, **RuP**-TiO_2 nanocrystals (0.2 mg/mL) was applied to QCM-D crystals. RuP-TiO_2 nanocrystal binding was performed in buffer (25 mM EDTA at pH 7.4). The plots shown are representative of triplicate experiments. (B) AFM height images of RuP-TiO_2 nanocrystals on the SAM-modified gold electrode, and the image is 1 µm × 1 µm and is displayed on a 10 nm z-scale. All gold surfaces used in this study are modified with 8-OH/8-NH_3^+ (80/20).

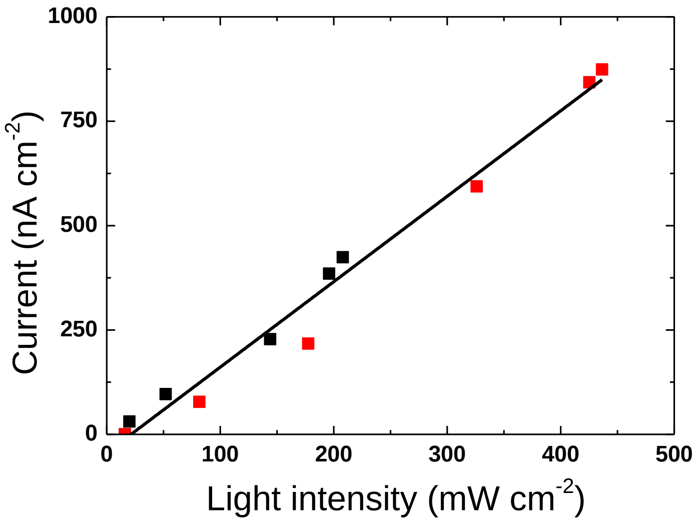

Figure S6: Measured photocurrent of TiO_2/MtrC conduit system onto SAM-modified gold electrode at 0.4 V vs SHE as a function of the light intensity. Black and red data points are from independent experiments. The line is a linear fit to all data.

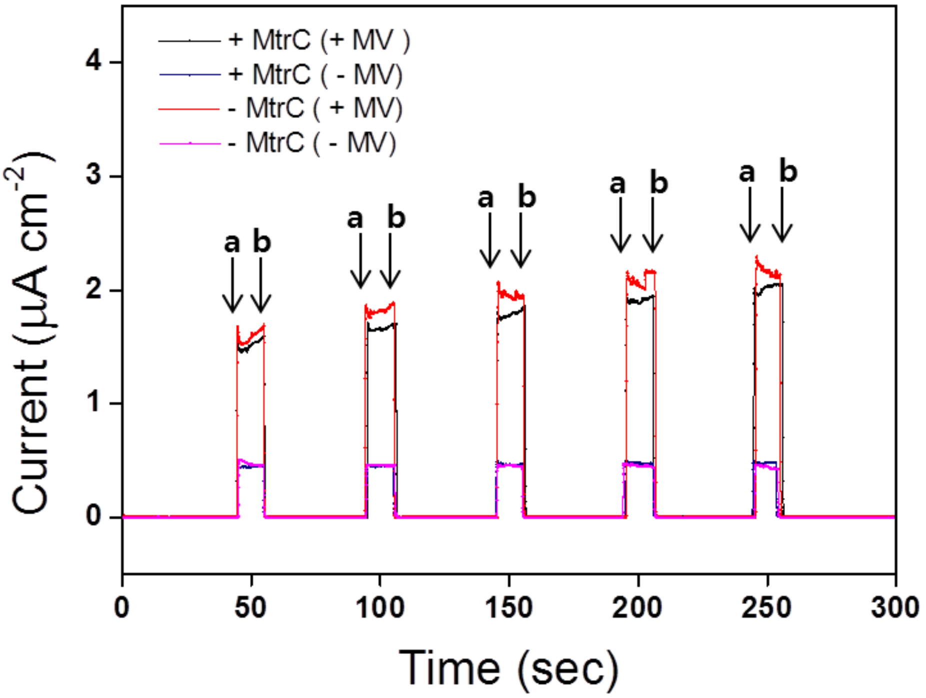

Figure S7. Measured photocurrent of TiO_2/MtrC conduit system and TiO_2 only system onto SAM-modified gold electrode at 0.4 V vs SHE. The photocurrent was measured at 20 °C in 20 mM EDTA, pH 7.4, either in the absence and presence of 1 mM methyl viologen (MV^{2+}). The switch “ON” (illumination on)-marked (a); switch “OFF”-marked (b)
